# Effects of different parenting styles on the physical health of Chinese university students

**DOI:** 10.3389/fpubh.2024.1433538

**Published:** 2024-08-27

**Authors:** Minghao Liu, Jingping Li, Sai Chang, Yi Liang, Zheng Wang

**Affiliations:** ^1^School of Physical Education, Soochow University, Suzhou, China; ^2^Soochow College, Soochow University, Suzhou, China; ^3^Admissions and Employment Office, Soochow University, Suzhou, China

**Keywords:** physical health, parenting styles, physical fitness, university student, PBI, influencing factors, China

## Abstract

**Background:**

Annual declines in university students’ physical health have an impact on social stability and the nation’s long-term growth. Parenting style, which is crucial to a child’s growth and development, may have a big influence on physical health. This study delves into the effects of different parenting styles on the physical fitness of Chinese college students under gender differences.

**Methods:**

Through random allocation and stratified sampling methods, this study comprehensively investigated 3,151 undergraduate students (male = 1,365; female = 1786) with an average age of 18.44 years (SD = 1.46), from a university in Jiangsu Province, China. Parenting styles of college students were evaluated by the Parental Bonding Instrument (PBI). Physical fitness tests were based on the Chinese National Student Physical Fitness Standards including body mass index, lung capacity, standing-long-jump, bent-leg-sit-up, seated forward bend, pull-ups, 50 m sprint, and 800/1,000 m run. Further investigations focused on the relationship between parenting styles and physical health by statistical analysis methods such as Pearson correlation and multiple linear regression.

**Results:**

Significant differences were identified in gender, family members, and academic majors in most of the correlation indicators between different parenting styles and physical health among college students. Further analysis showed that the parenting styles of democratic and authoritative mothers and democratic fathers were more conducive to the promotion of physical health among female university students. The combination of a democratic fathering style and a permissive mothering style is considered an ideal parenting model for male students.

**Conclusion:**

This study confirmed that different parenting styles have a significant impact on the physical health of college students. Positive parenting styles may improve physical health, while negative ones are likely to have adverse effects, especially among female students. It is also important to notice differentiated parenting styles with respect to male and female university students. Therefore, more attention should be raised on parenting styles to enhance physical health of the student population.

## 1 Introduction

College students are the future of a nation, and their physical and mental well-being is not only essential to their own personal development but also to the long-term progress, social peace, and stability of the nation. However, multiple studies have shown that the level of physical health among college students has been declining yearly (1, 2). This trend may not only increase the risk of cancer (3) but also reduce their sense of happiness and even increase the risk of depression and anxiety (4). It is paramount to emphasize that college represents a pivotal juncture in students’ lives, marking their transition from adolescence to adulthood (5), which is an important period for their psychological maturity and the formation of healthy lifestyles and behaviors (6). They face serious obstacles to their physical and mental health throughout this phase as they adjust to their new surroundings and deal with the demanding demands of their academic work. Therefore, it is particularly important to explore the factors that affect the physical health of college students and find practical solutions.

Physical health serves as the foundation for an individual’s overall development. It is not solely related to the physical condition of an individual (7); rather, it is also a crucial indicator of the overall health status of a country’s citizens (8). Good physical health not only means that an individual possesses more robust physical activity capabilities but also prevents critical non-communicable diseases such as cardiovascular diseases. At the same time, it has a profound impact on improving learning and work efficiency (9). In addition, student physical health issues have always been a focus of scholars’ attention. Regarding the trends in physical health, Pribis’ research indicated that the physical health level of American college students has been gradually and slowly declining over the past 13 years (10). In a similar study, Dong observed a comparable trend in the physical health of Chinese college students, indicating a decrease in their physical health levels from 2013 to 2019 (2). As for the influencing factors, certain research has indicated a positive association between the mental and physical health of children and adolescents (11, 12). Furthermore, a detailed analysis of relevant indicators related to physical health underscores the positive correlation between mental health and cardiopulmonary health among middle school students (13, 14). In terms of studying specific physical health-related indicators, longitudinal studies have pointed out that the COVID-19 pandemic has affected the BMI and obesity detection rates among adolescents (15). Other studies have delved into the driving forces for muscles among French adolescents within the sociocultural framework (16). However, despite the relatively deep understanding of physical health from previous studies, there is a lack of research focusing on the factors that affect physical health and related indicators of college students, a particular group.

Among the numerous factors that affect physical health, parental rearing styles are an essential factor that cannot be ignored. Parents are the first educators of their children, and their rearing styles directly influence the growth and development of their children (17). In particular, the family environment, as a primary socializing agent, holds significant influence adolescents’ exercise behaviors and habits (18, 19). Families provide the foundation for learning and adopting health behaviors (20). Extensive research has demonstrated the impact of the family environment on the formation of exercise habits among adolescents (21). For example, factor such as family support (22–24), parental role modeling (25, 26), and the availability of resources and opportunities for physical activity within the family context (23) have been found to be crucial in influencing adolescents’ exercise behaviors. While, healthy habits are often cultivated from an early age and gradually consolidated during childhood. However, the consolidation or potential transformation of these habits becomes increasingly challenged during adolescence and early adulthood. Specifically, the college student population at the dawn of early adulthood stands at a delicate balance between the shelter of their family and the independence of life. This period represents a pivotal turning point in personal growth, as well as a crucial juncture to explore how early familial environments continue to influence and potentially reshape their physical activity behaviors, thereby impacting their overall physical fitness and health status.

Multiple studies have confirmed that parental rearing styles have a significant predictive effect on children’s psychological growth, health status, and future behavioral problems. For instance, Ebrahimi and others found that there is a correlation between parental rearing styles and childhood depression (27). Specifically, the supervisory characteristics exhibited by authoritative parents have a protective effect on adolescents’ participation in cyberbullying, while authoritarian styles may increase the risk of children becoming targets of cyberbullying (28). Based on this, Fatima and others pointed out that authoritative rearing styles are positively correlated with prosocial behavior. In contrast an authoritarian style exhibited by mothers is negatively correlated with prosocial behavior (29). Parental rearing styles also play an essential role in health conditions. Jago’s research showed that parents’ permissive attitudes are associated with higher levels of physical activity in children. In contrast parents’ encouragement and support have positively impact on boys’ and girls’ physical activities, respectively (30). However, Johnson’s further analysis found that permissive child-rearing styles are associated with environments that are more likely to lead to obesity, which partly explains the family factors behind childhood obesity issues (31). Additionally, Kakinami and his colleagues’ research discovered that authoritarian parenting styles are also associated with an increased risk of childhood obesity (32). Despite various parenting styles, most studies agree that authoritative parenting styles are less likely to contribute to obesity (33–35). This underscores the importance of authoritative parenting in preventing childhood obesity and highlights the negative impact of authoritarian parenting on children’s health outcomes.

Previous research has established a foundation for understanding the relationship between parental rearing styles and the physical health of children and adolescents. However, these studies have primarily focused on childhood or adolescent stages, with a relative scarcity of in-depth research on the specific group of college students, and the underlying mechanisms remain unclear. Notably, during the college phase, parents’ rearing styles may still exert a significant, albeit subtle, influence on their children’s physical health. Additionally, existing research often neglects the impact of gender differences in this relationship. Given the profound differences between males and females in physiology, psychology, and social roles, these disparities will likely result in varying responses and adaptation patterns to parental rearing styles. Therefore, this study comprehensively employed questionnaires and physical fitness tests to collect and analyze relevant data, aiming to delve deeper into the specific impacts of different rearing styles on the physical health of college students and further explore the gender-specific differences in these impacts.

The objectives of this study were to (1) comprehend the overall level and distribution of different parenting styles among college students, (2) analyze the current status and influencing factors of physical health among college students with gender differences taken into account, and (3) explore the potential impacts and underlying mechanisms of different parenting styles on physical health, considering the gender disparities. In summary, our goal was to achieve a deeper insight into the intricate interplay between parenting styles and physical health among college students, as well as to explore potential gender-based variations in these relationships.

## 2 Materials and methods

### 2.1 Study design

The study collected data at a university in Jiangsu Province, China. Before the study, we informed the participants about the research objectives and procedures. The participants freely decided whether to participate or withdraw from the investigation. We obtained their consent and provided those interested in learning more about the study’s details with a basic overview upon completion. The inclusion criteria for this study were as follows: Firstly, a simple random sampling technique was employed to select 13 secondary colleges (out of 26) from Soochow University. Secondly, we adopted a cluster sampling approach to identify undergraduate majors across the selected secondary colleges as the subjects of our research. Thirdly, regarding data collection for the Parental Bonding Instrument questionnaire, approximately 2 weeks before the fitness assessment test, an online questionnaire was distributed to the participants. The questionnaire was designed to be completed independently and objectively by the students themselves to obtain first-hand information about parenting styles, without the need for direct parental involvement. A total of 3,630 freshmen completed and submitted the online questionnaire, originating from various provinces and cities across China, including Jiangsu, Shanghai, Henan, Shandong, and others. Fourth, physical fitness test: Physical fitness tests were based on the Chinese National Student Physical Fitness Standards including body mass index, lung capacity, standing-long-jump, bent-leg-sit-up, seated forward bend, pull-ups, 50 m sprint, and 800/1,000 m run. To ensure the validity of the study data, participants were excluded for any one of the following conditions: (1) the presence of significant physical or psychological illnesses, (2) incomplete participation in all physical fitness testing items, and (3) abnormally short questionnaire completion time or arbitrary responses (such as providing identical answers). A total of 3,151 college students with an average age of 18.44 years (43.32% male and 56.68% female) were ultimately included in the analysis. The university’s ethics committee granted the ethical approval for this study (ID: ECSU-2019000154).

### 2.2 Parental bonding instrument scale

The Parental Bonding Instrument (PBI), initially developed by Parker et al., is a well-established psychological measurement tool (36). On this foundation, Yang and his colleagues attentively fine-tuned and revised the questionnaire to better align it with the cultural nuances and growing context of Chinese college students (37). This adapted version not only retains the core elements of the original questionnaire but also integrates unique aspects of Chinese family culture and educational beliefs, making it more relevant to the actual situation of Chinese college students. As a result, it provides a more accurate and practical measurement tool for this study.

The scale comprises two versions: the PBI-M (for mothers) and the PBI-F (for fathers). Both versions cover three dimensions: care, encouragement of autonomy, and control. Each version contains 23 items, scored using a Likert 4-point scale, ranging from “0” (strongly disagree) to “3” (strongly agree). The Cronbach’s α coefficients and split-half reliability coefficients for the three dimensions of the PBI-M were 0.846/0.830 for motherly care, 0.806/0.727 for motherly encouragement of autonomy, and 0.745/0.661 for motherly control. Similarly, in the PBI-F, the coefficients were 0.858/0.844 for fatherly care, 0.822/0.748 for fatherly encouragement of autonomy, and 0.752/0.689 for fatherly control. The test–retest correlation coefficients range from 0.746 to 0.941, indicating good reliability and validity. In conclusion, the scale is reliable and practical in measuring parenting styles of Chinese college students.

### 2.3 Physical health assessment

The physical health test is based on the Chinese National Student Physical Fitness Standards, a reliable and valid physical fitness test used by researchers (38). The physical fitness test consists of seven indicators measuring, in order, height, weight, lung capacity, seated forward bend, 50-meter sprint, standing-long-jump, pull-ups (male)/bent-leg-sit-ups (female) and 1,000-meter run (male)/800-meter run (female). University physical education instructors are responsible for administering the fitness test.

#### 2.3.1 BMI

A height and weight tester (HK6800-ST, China) measured the height and weight of the subjects. During the test, subjects were barefoot and wore only thin clothing. Body Mass Index (BMI) is calculated by dividing weight by height squared (kg/m^2^), using the WHO definition of BMI, with scores equal to and higher than 28 (kg/m^2^) defined as obese, 24 to 27.9 as overweight, 18.5 to 23.9 as normal weight, and less than 18.5 as low weight (39).

#### 2.3.2 Lung capacity

The lung capacity was assessed by means of the FHL-II spirometer (New East Huateng Co., Ltd., Beijing, China). After the first maximal expiration, participants rested for 20 s before the second test and the best performance was recorded.

#### 2.3.3 800/1000-m run

The participants run the 1,000 m test for males and the 800 m test for females on a 400 m track. Before the test, students are required to do a full warm-up and then they run in groups of 10–12 using a standing start.

#### 2.3.4 Standing long jump

The standing long jump is tested on a sand pit with a sand surface flush with the ground or on a flat surface of soft soil using a measuring tape. The tester was required to jump with both feet at the same time and measure the horizontal distance from the trailing edge of the starting point to the trailing edge of the nearest landing point. Each student was allowed three jump attempts, with the longest distance selected to be recorded in centimeters.

#### 2.3.5 50-m sprint

The 50-m sprint test is conducted on an athletic field. The subjects were organized into groups of 2–3 individuals and participated in a race with a standing start, when the testers heard the start signal, they immediately took off and ran as hard as they could to the finish line. Each participant was allowed two attempts and the best performance was selected. Records were kept in seconds to one decimal place.

#### 2.3.6 Seated forward bend

The seated forward bend test was performed using an electronic sitting flexion-testing machine (Wanqing WTS-600, Shanghai, China). The test subject was asked to sit on the test plate with legs straight and feet flat on the test longitudinal plate, bend the upper body forward, extend the arms forward, and gradually push the Vernier forward with the tip of the middle finger until it could not be pushed forward. Recordings were made in centimeters to one decimal place.

#### 2.3.7 Pull-ups

Pull-ups are used to assess upper body muscle strength. The test was counted as pull-ups. The subject jumped and pulled with both hands on a bar. After standing still, the subject pulled with both arms simultaneously. All male students took the test.

#### 2.3.8 Bent-leg-sit-up

Subjects was instructed to lie on a mat with their knees bent at 90 degrees, lift their upper body and touch their knees with their elbows. The number of bent-leg sit-ups completed within 1 min was recorded.

The total score of physical fitness consists of the sum of the product of the scores and weights of each single indicator and is 120 points. Students are graded according to their total score: ≥90.00 as excellent; 80.0–89.9 as good; 60.0–79.9 as passing; and ≤ 59.9 as failing. Total Physical Fitness Score = 15%*BMI + 15%*Lung Capacity +20%*50-meter Sprint +10%*Seated Forward Bend +10%*Standing Long Jump +10%*Males’ Pull-ups/Females’ Bent-leg and Sit-up +20%*Males’ 1,000-meter run/Females’ 800-meter run.

### 2.4 Statistical analysis

Descriptive and inferential statistical analyses were conducted using IBM SPSS Statistics 26. In order to examine differences in parenting styles and physical health across gender, place of birth, family members, and subject specialties, we employed a t-test. We further conducted a Pearson correlation analysis to delve into the correlation between diverse parenting styles and physical health. Significantly associated parenting style factors were subsequently included as predictor variables in a multiple regression analysis, with physical health indicators as the dependent variables. Additional covariates, such as age, geographical origin, duration of testing, and student ID, were also considered in the analysis. Three hierarchical models were constructed: Model 1 incorporated different parenting styles, Model 2 added duration of testing and student ID variables to Model 1, and Model 3 included age and geographical origin variables based on Model 2. Model 3 served as the primary analytical model throughout the study, gradually revealing the combined impact of these factors on physical health indicators. Additionally, we conducted a one-way ANOVA to further analyze and compare the different types of parenting styles and their respective associations with physical health indicators in a post-hoc manner. We graphically represented the selected results using Graph Pad Prism 8. The statistical significance level was set at 0.05. Given the inherent differences in physical fitness testing requirements, items, and scoring criteria between males and females, this study conducted separate analyses for both genders while acknowledging these gender-specific differences in physical fitness.

## 3 Results

### 3.1 Socio-demographic and parenting style characteristics

The study encompassed 3,151 participants, with 1,365 males (43.32%) and 1,786 females (56.68%). Appendix 1 provides an overview of the demographic characteristics of the participants, including gender, family member, place of origin, and academic major, along with an analysis of the scores of different parenting styles among these four subgroups of students. The study of parenting styles among university students revealed several notable findings. Female students scored significantly higher regarding mothers’ encouragement of autonomy and fathers’ affection than male students. Moreover, students from single-child families exhibited considerably higher scores in mothers’ affection, mothers’ control, fathers’ affection, and fathers’ encouragement of autonomy than those from non-single-child families. Students originating from urban areas also scored significantly higher in mothers’ affection, mothers’ encouragement of independence, fathers’ affection, and fathers’ encouragement of independence than those from rural areas. No significant differences were observed in the scores of different parenting styles among students of various academic majors. These findings provide valuable insights into the diverse parenting styles and their potential influence on students’ development. Understanding these patterns can inform educational practices and strategies to promote studevnt well-being and success.

### 3.2 Socio-demographic and physical health characteristics

Appendix 2 summarizes the sociodemographic characteristics of the participants, including gender, family member, place of origin, and academic major, along with an analysis of their physical fitness. Significant differences in physical health were observed among university students.

Among female students, those from single-child families exhibited significantly higher scores in lung capacity, 50-m sprint, and bent-leg-sit-ups than those from non-single-child families. Conversely, non-single daughters performed better in standing long jump, 800-meter run, and seated forward bend, with significant differences observed. Female students hailing from urban areas significantly outperformed their rural counterparts in lung capacity, 50-meter sprint, sit-ups, cardiopulmonary fitness, and overall physical fitness. However, rural female students demonstrated superior performance in standing long jump, with significant differences. Female students majoring in natural sciences outperformed those in social sciences in the 50-meter, standing long jump, 800-meter run, bent-leg-sit-ups, cardiopulmonary fitness, and overall physical fitness, with notable differences.

For male students, those from single-child families showed significantly higher lung capacity scores compared to non-single sons. On the other hand, non-single sons demonstrated better performance in BMI, standing long jump, 1,000-meter run, seated forward bend, pull-ups, and overall physical fitness, with significant differences. Male students from urban backgrounds scored higher in lung capacity than those from rural areas. In contrast rural male students excelled in sitting forward bends and pull-ups, with notable differences. Male students majoring in natural sciences exhibited superior lung capacity, pull-ups, cardiopulmonary fitness, and overall physical fitness compared to those in social sciences, with substantial differences observed.

### 3.3 Correlation and multiple linear regression analysis

Correlation analysis revealed that various parenting styles were associated with physical fitness indicators, with significant gender differences (see Appendix 3). The results of the multiple linear regression model (Model 3) demonstrated that different parenting styles had significant relationships with multiple indicators of physical health. Notably, the father’s controlling factor was not associated with physical health measures.

For instance, among female students, the mother’s nurturing factor had a significant positive predictive effect on lung capacity (*β* = 0.104, *p* < 0.01). This indicated that for every one-point increase in the mother’s nurturing factor, there was a corresponding increase of 0.104 points in lung capacity. Conversely, for male students, the father’s encouraging autonomy factor had a significant negative predictive effect on pull-ups (*β* = −0.039, *p* < 0.05). This meant that a one-point increase in the father’s encouraging autonomy factor resulted in a decrease of 0.039 points in the male students’ pull-up performance.

Tables 1, 2 offer a comprehensive examination of the significant predictive correlations between parenting factors, encompassing nurturing, encouragement of autonomy, controlling behavior, and physical health, while specifically highlighting the gender-specific differences in these relationships. These findings underscore the intricate connections between parenting styles and individual physical fitness, highlighting the importance of considering gender-specific factors in understanding and fostering optimal physical health.

**Table 1:** Multiple linear regression analysis of parenting styles and college students’ physical fitness scores for female students (*N* = 1786).

Implicit variable	Care		Autonomy		Over-protection	
	*β* (95%CI)	*p*	*β* (95%CI)	*p*	*β* (95%CI)	*p*
Mothers’ parenting styles						
Lung capacity	**0.104 (0.015, 0.061)**	**<0.01**	**−0.083 (−0.078, 0.007)**	**<0.05**	−0.056 (−0.086, 0.006)	0.09
50-m sprint	**0.121 (−0.031, 0.100)**	**<0.01**	**0.128 (0.044, 0.149)**	**<0.01**	0.045 (−0.020, 0.117)	0.17
Standing-long-jump	0.045 (−0.004, 0.024)	0.53	**0.088 (0.005, 0.049)**	**<0.05**	0.005 (−0.026, 0.031)	0.88
800-m run	0.047 (−0.010, 0.066)	0.15	0.069 (−0.001, 0.116)	0.06	**−0.088 (−0.180, −0.027)**	**<0.01**
Bent-leg-sit-ups	**0.105 (0.011, 0.042)**	**<0.01**	0.033 (−0.013, 0.036)	0.35	0.007 (−0.029, 0.035)	0.84
Physical fitness score	**0.137 (0.092, 0.244)**	**<0.01**	**0.108 (0.066, 0.300)**	**<0.01**	−0.046 (−0.262, 0.042)	0.16
Fathers’ parenting styles						
BMI	**0.085 (0.005, 0.037)**	**<0.01**	−0.020 (−0.033, 0.018)	0.57	0.014 (−0.027, 0.043)	0.66
Lung capacity	−0.043 (−0.035, 0.007)	0.18	**0.130 (0.031, 0.098)**	**<0.01**	0.031 (−0.023, 0.069)	0.32

Bold indicates that the value is significant.

**Table 2:** Multiple linear regression analysis of parenting styles and college students’ physical fitness scores for male students (*N* = 1,365).

Implicit variable	Care		Autonomy		Over-protection	
	*β* (95%CI)	*p*	*β* (95%CI)	*p*	*β* (95%CI)	*p*
Mothers’ parenting styles						
1,000-m run	−0.019 (−0.060, 0.035)	0.61	**0.222 (0.117, 0.248)**	**<0.01**	0.040 (−0.040, 0.127)	0.31
Pull ups	−0.020 (−0.028, 0.016)	0.59	**0.158 (0.030, 0.091)**	**<0.01**	−0.002 (−0.040, 0.038)	0.96
Physical fitness score	−0.002 (−0.121, 0.113)	0.95	**0.185 (0.216, 0.539)**	**<0.01**	−0.035 (−0.300, 0.110)	0.36
Fathers’ parenting styles						
Pull ups	0.009 (−0.010, 0.029)	0.35	**−0.039 (−0.069,-0.008)**	**<0.05**	−0.018 (−0.059, 0.023)	0.39

Bold indicates that the value is significant.

### 3.4 Comparison of physical health outcomes among college students with different parenting styles

Using the scores of nurturing and controlling factors, we categorized parenting styles into four types based on the mean values: Authoritative type (T1: high caring, high controlling), Autocratic type (T2: low caring, high controlling), Democratic type (T3: high caring, low controlling), and *Laissez-faire* type (T4: low caring, low controlling) (40). A one-way ANOVA was conducted to observe differences in interpersonal reactivity indices among college students with different parenting styles, considering gender differences.

**Figure 1:**
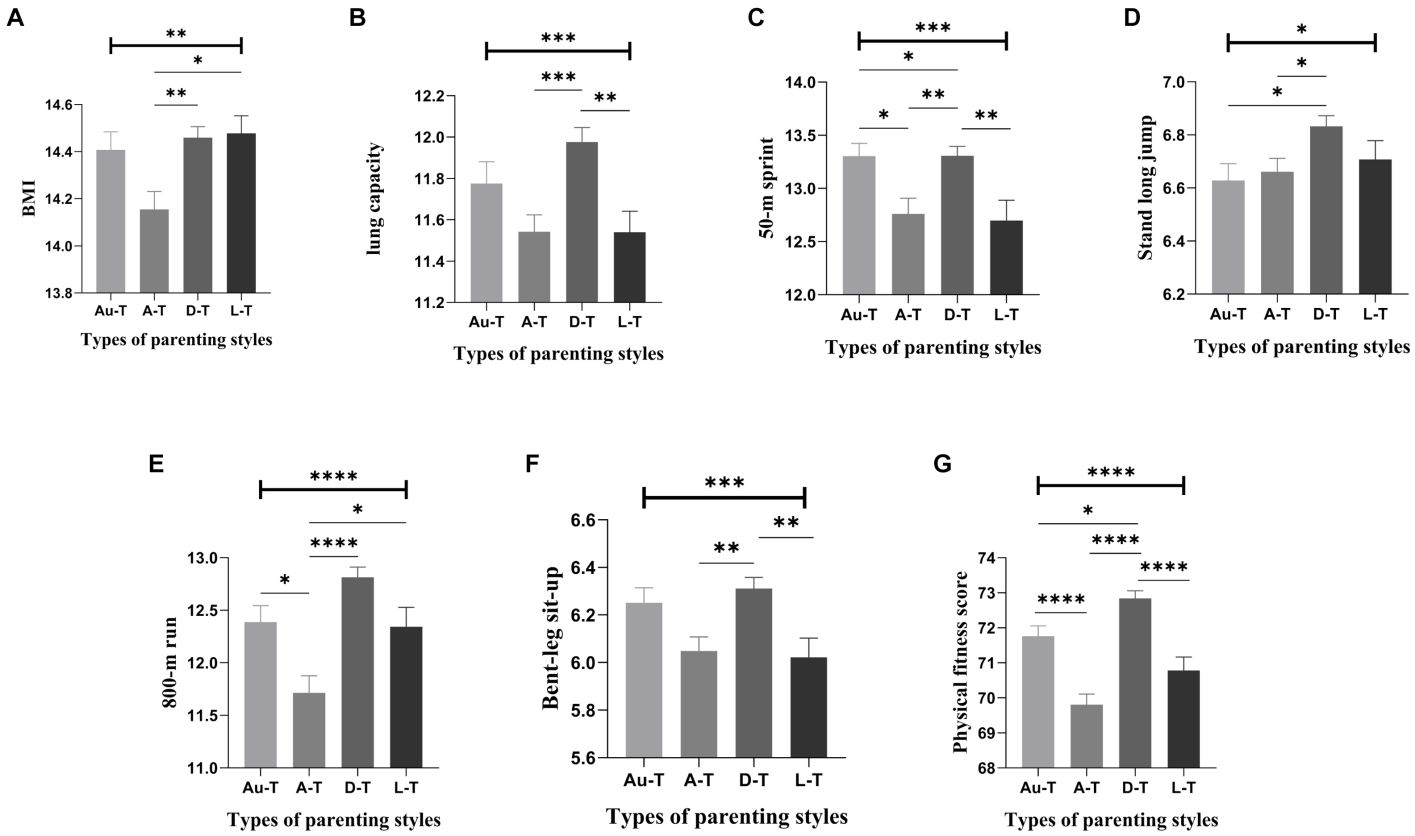
Comparison of scores related to females’ physical fitness across different types of mother’s parenting styles (Mean ± SE). Error bars represent the standard error(SE); Au-t, Authoritative type; A-T, Autocratic type; D-T, Democratic type; L-T, Laissez-faire type; *p < 0.05; **p < 0.01; ***p < 0.001; ****p < 0.0001.

**Figure 2:**
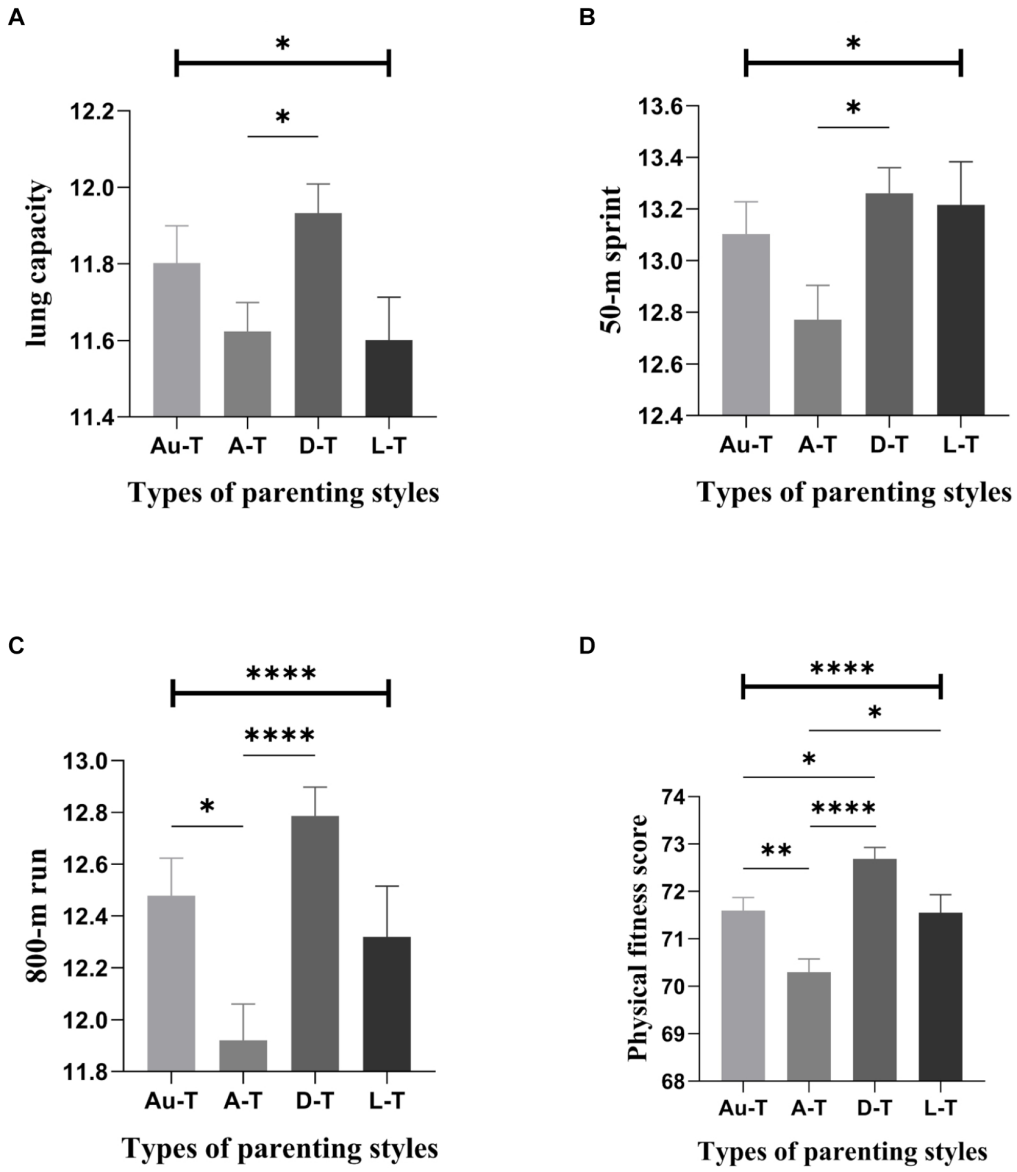
Comparison of scores related to females’ physical fitness across different types of father’s parenting styles (Mean ± SE). Error bars represent the standard error(SE); Au-t, Authoritative type; A-T, Autocratic type; D-T, Democratic type; L-T, Laissez-faire type; *p < 0.05; **p < 0.01; ****p < 0.0001.

Further post-hoc analysis among female students revealed significant intergroup differences in BMI, lung capacity, 50-meter run, standing long jump, 800-meter run, sit-ups, cardiopulmonary fitness, and overall physical health based on different parenting styles. Pairwise comparisons of mother’s parenting styles showed, for instance, that students with a *laissez-faire* style had the highest BMI scores, substantially higher than those with an autocratic style. Students with a democratic style had significantly higher lung capacity scores than those with autocratic and *laissez-faire* styles, with no significant difference from the authoritative style (Figure 1). Pairwise comparisons of father’s parenting styles indicated, for example, that students with a democratic style had the highest lung capacity scores, significantly higher than those with an autocratic style, with no significant difference from the authoritative and *laissez-faire* styles. Similarly, students with a democratic style scored the highest in the 50-meter run, significantly higher than those with an autocratic style, and there were no significant differences compared to the authoritative and *laissez-faire* styles (Figure 2).

**Figure 3:**
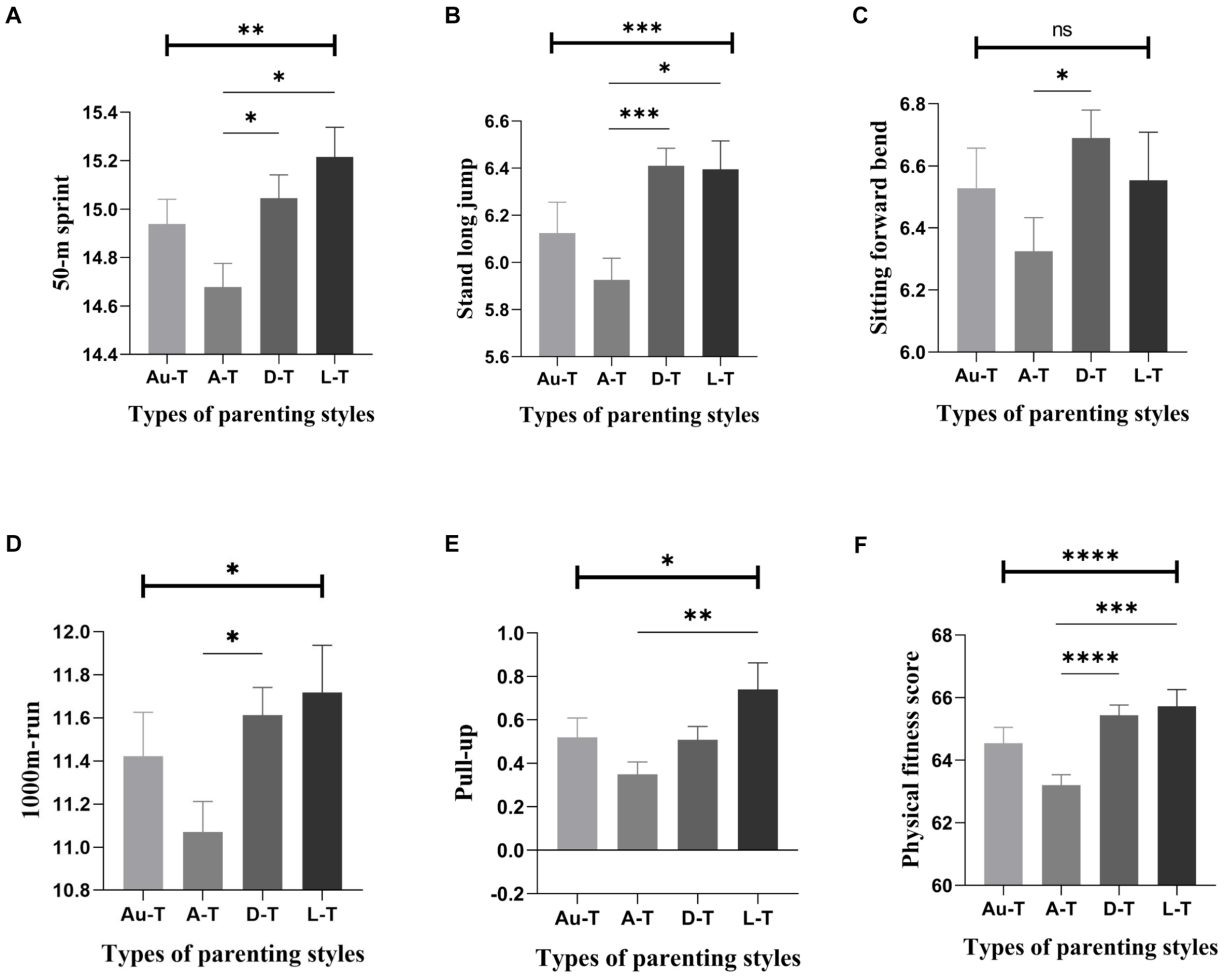
Comparison of scores related to males’ physical fitness across different types of mother’s parenting styles (Mean ± SE). Error bars represent the standard error(SE); Au-t, Authoritative type; A-T, Autocratic type; D-T, Democratic type; L-T, Laissez-faire type; *p < 0.05; **p < 0.01; ***p < 0.001; ****p < 0.0001.

**Figure 4:**
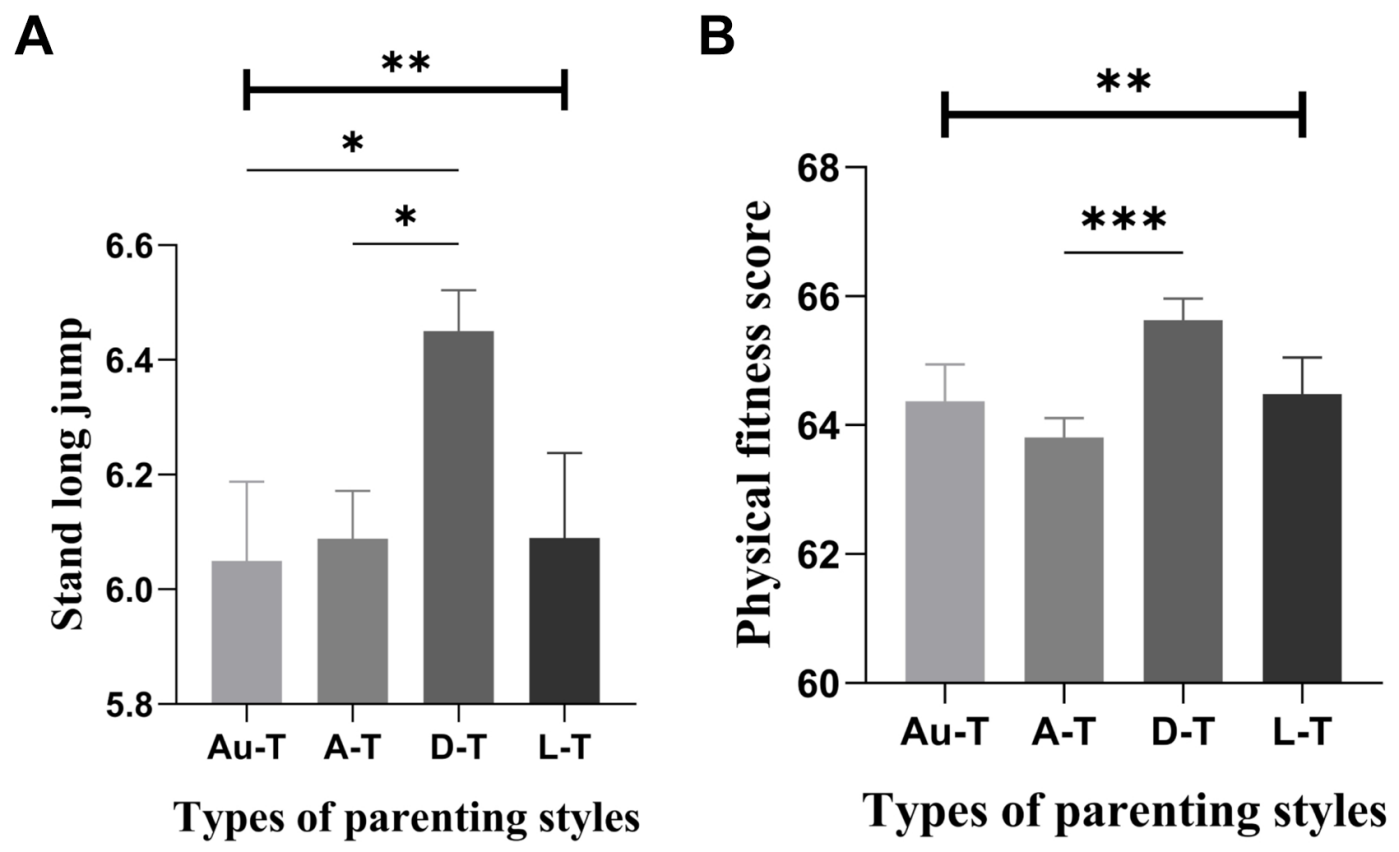
Comparison of scores related to males’ physical fitness across different types of father’s parenting styles (Mean ± SE). Error bars represent the standard error(SE); Au-t, Authoritative type; A-T, Autocratic type; D-T, Democratic type; L-T, Laissez-faire type; *p < 0.05; **p < 0.01; ***p < 0.001.

Further post-hoc analyses of the males showed that there were significant differences between the different parenting styles in the 50-meter run, standing long jump, 800-meter run, pull-ups, and overall physical fitness. Pairwise comparisons of mothers’ parenting styles indicated that students with a *laissez-faire* style scored the highest in the 50-meter run, significantly higher than those with an autocratic style. Still, there were no significant differences from those with an authoritative or democratic style. Notably, students with a democratic style also performed significantly better than those with an autocratic style in the 50-meter run. Regarding standing long jump, students with a democratic style achieved the best results, significantly higher than those with an autocratic style, with no significant differences from those with an authoritative or *laissez-faire* style. However, students with a *laissez-faire* style also performed significantly better than those with an autocratic style in this metric (Figure 3). Comparisons of father’s parenting styles showed that students with a democratic style excelled in the standing long jump, scoring significantly higher than those with both an authoritative and autocratic style. Still, there were no significant differences from those with a *laissez-faire* style (Figure 4).

## 4 Discussion

This study specifically targeted Chinese university students, delving into the impact of various parental nurturing styles on their physical health, with a particular focusing on gender-specific manifestations. Through a comprehensive application of scientific methodologies such as Pearson correlation analysis, multiple linear regression, and one-way ANOVA, we arrived at unequivocal findings: parental nurturing styles exert varying degrees of influence on the physical health of university students. Notably, authoritative and democratic styles of nurturing styles positively correlate with promoting physical health among students, whereas authoritarian and permissive styles tend to have negative impacts. This trend is even more pronounced among female students. On these revelations, our research underscores the crucial role of improving parental nurturing styles in enhancing the physical health of university students. We urge all sectors of society, particularly educational institutions and families, to increase their attention and guidance towards parental nurturing styles. By actively guiding and assisting parents in adopting more scientific and reasonable nurturing approaches, we can create favorable conditions for the comprehensive development of students’ physical health. Furthermore, given the gender differences observed, it is imperative to develop and implement more targeted intervention measures, thus effectively promoting the enhancement of university students’ physical health.

### 4.1 Differences in physical fitness of college students

A descriptive analysis conducted has revealed noteworthy disparities in physical health among college students, particularly in terms of gender differences and the influence of various factors such as family composition, hometown type, and academic major. Consistent with the observations made by Rodriguez-Ayllo and others, these differences underscore the impact of societal, familial, and individual motivations on the fluctuations in individuals’ physical activity levels (41). In addition, there were significant differences in the physical performance of different categories of students. For example, non-only children tend to have significantly higher standing long jump scores compared to only children. Conversely, male students from urban backgrounds tended to score lower in pull-ups than those from rural backgrounds. These findings provide further evidence of the vital role of acquired physical activity in maintaining optimal health and well-being (7).

A recent study has revealed that the obesity risk among urban students and only children in China is gradually increasing. In contrast, non-only children and rural students tend to have better physical health, which aligns with most findings in our study (42). However, there are some inconsistencies with previous research. For instance, students from single-child families, regardless of their gender, demonstrated notably higher lung capacity scores in comparison to those who have siblings in their households. This can be attributed to urban–rural differences in physical activity are not static, and their direction and significance may vary depending on the study population, time frame, and measurement methods (43). Furthermore, our study revealed that female students hailing from urban backgrounds possessed significantly higher levels of physical health compared to their counterparts from rural areas, where as among male students, no significant disparity was observed. This trend can be explained by the recent government efforts to promote new urbanization, which has improved the living standards of rural residents and narrowed the urban–rural gap (44). In addition, students majoring in natural disciplines generally had higher levels of physical fitness than students majoring in social disciplines among male and female students, suggesting that differences in disciplinary specialization may affect levels of physical fitness.

### 4.2 Differences in parental styles of college students

Descriptive analysis further revealed significant differences in parental nurturing styles across gender, family composition, and geographical background. Specifically, female students whose mothers encouraged an autonomous style scored significantly higher than male students and female students whose fathers displayed a nurturing style also received higher scores than male students. T This trend can be attributed to the differing expectations parents often hold for their sons and daughters, which results in the adoption of varying educational approaches for each gender. Additionally, boys and girls may perceive parenting behaviors differently, resulting in different responses, particularly during adolescence (45). Regarding family composition, parents of only children scored significantly higher in terms of caring. At the same time, mothers’ controlling styles and fathers’ encouragement of autonomy were also more pronounced compared to those of non-only children. This trend can be attributed to the fact that in families with only one child, parents provide more decadent emotional warmth and understanding, offering increased emotional support that results in more positive psychological experiences for the child (2). However, as parents usually place all their hopes and expectations on the only child, these children often face multiple pressures. As a result, parents may exhibit overprotectiveness towards their child, frequently exhibiting excessive concern for their safety and well-being. This tendency often manifests in a pattern of overprotective parenting, where parents tend to take on tasks and decisions for their child. Regarding to geographical origins, parents of urban students demonstrated notably higher scores in nurturing their children and encouraging autonomy compared to parents residing in rural areas. This trend can be attributed to the differing effects of nurturing styles based on families’ socio-economic status (45, 46). Typically, urban parents enjoy higher levels of education and economic income than rural parents, allowing them to invest more energy and resources in their children’s education, which manifests as increased concern and attention. Notably, this study revealed no significant disparities in parental nurturing styles across various academic disciplines. This discovery provides us with a deeper understanding of the influencing factors of parental caring styles, yet further research is needed to unravel the complex mechanisms behind them fully.

### 4.3 The relationship between parenting styles and females’ physical health

The findings for female students revealed several interesting correlations. Specifically, a mother’s caring factor positively correlated with lung capacity, 50-meter dash performance, sit-ups, and overall physical health score. However, the mother’s encouragement of autonomy exhibited a negative correlation with lung capacity but a positive correlation with performance in the 50-meter dash and standing long jump. Conversely, the mother’s controlling factor negatively correlated with the 800-meter run. On the other hand, a father’s caring factor is positively related to BMI. Notably, the father’s encouragement of autonomy is positively associated with lung capacity.

BMI, a commonly used indicator to measure obesity, is influenced by various factors such as dietary habits and exercise routines. When fathers show more nurturing care towards their daughters, it often leads to a heightened sense of emotional support and security, which strongly increases the desire for healthy food (47). Lung capacity, an essential metric for assessing respiratory function (48), is directly linked to an individual’s physical fitness and endurance (49). Notably, lung capacity significantly impacts a girl’s ability to engage in daily activities and perform in various running challenges (50). Interestingly, although factors like maternal nurturing and parental encouragement of autonomy were identified as significant predictors of lung capacity levels, the study did not uncover a direct correlation between these factors and performance in the 800-meter endurance run. This finding indicates that while parental nurturing styles may positively or negatively influence a female’s respiratory function, these effects do not necessarily translate into direct gains or losses in long-distance endurance running performance. The 50-meter sprint is a crucial indicator of speed, reflecting the ability to move quickly on the ground or with the limbs (51). Both maternal caring and encouragement of autonomy positively correlate with scores in the 50-m sprint, suggesting that a mother’s positive parenting approach positively impacts her daughter’s performance in this short-distance event. When mothers shower their daughters with love and encouragement, it consistently cultivates a positive emotional landscape, fostering a sense of well-being and resilience. Negative emotions, on the other hand, can slow down movement speed, especially evident in speed tests like the 50-meter sprint (52). Therefore, females who possess positive emotions are more likely to achieve excellent results in speed events. The bent-leg-sit-ups test is a classic assessment of abdominal muscle strength and endurance, serving as a reliable indicator of the robustness of the core musculature. This test not only reflects the strength of the core muscles but also correlates closely with overall physical health and performance (53). The strength of abdominal muscles is pivotal in daily activities and sports, providing essential support and stability to the lumbar vertebrae and pelvis (54). Additionally, it significantly enhances trunk extension, hip joint function, and overall bodily strength (55). Consequently, under the influence of maternal nurturing, female students may possess superior physical fitness. The standing long jump, a classic test for assessing lower-body explosive power and overall body coordination, exhibits a positive correlation with motherly encouragement for autonomy. This suggests that when mothers encourage girls to think independently, make autonomous decisions, and grant them sufficient freedom to explore and develop their interests and abilities in daily life, girls tend to achieve better results in the standing long jump. On the contrary, a negative correlation is observed between maternal control factors and performance in the 800-meter run. This finding reveals the complexity of how motherly parenting styles influence the physical development of students. Under strict motherly control, girls may psychologically develop a resistance to sports, thereby affecting their performance in long-distance running.

Finally, the overall physical health score serves as a comprehensive evaluation of an individual’s overall well-being, encompassing various aspects of physical fitness and functional indices. In our study, the positive correlation between factors such as motherly care and encouragement for autonomy and the overall physical health score indicates that the nurturing and encouragement of a mother positively contribute to the overall health level of female students. This underscores the significance of positive psychological emotions in promoting holistic physical development (56). However, the study did not reveal a significant association between parental parenting styles and sitting forward bend performance. This suggests that the influence of parental nurturing on female athletic ability is not comprehensive but selective and specific. While certain aspects of physical performance may be influenced by parental factors, others may be less responsive to such influences.

To further observe the characteristics of physical health among college students reared under different parenting styles, the study analyzed and compared the differences in physical health among students who grew up under four typical parenting approaches (40). The results revealed that, based on different types of maternal parenting styles, individuals reared under a permissive style had significantly higher BMI scores compared to those reared under an authoritarian style. On the other hand, those reared under a democratic style exhibited the highest scores in lung capacity, 50-meter dash, 800-meter run, sit-ups, and overall physical health. When considering different types of paternal parenting styles, individuals reared under a democratic approach also demonstrated the highest scores in lung capacity, 50-meter dash, 800-meter run, and overall physical health. Conversely, among the various parenting styles, those reared under a predominantly authoritarian style tended to have the lowest scores in physical health-related indicators. From the perspective of female students’ physical health development, a democratic parenting style is considered an ideal approach, which contradicts previous research findings. Prior studies have suggested that an authoritative parenting style is the most beneficial, associated with improved physical and mental health outcomes in adulthood. However, our findings indicate that a democratic style, which emphasizes mutual respect and open communication between parents and children, may be more conducive to the overall well-being of female students. This observation suggests that different parenting styles may have varying impacts on individuals, depending on their gender and personal characteristics (57–60). The authoritarian type is one of the most detrimental parenting styles to the development of physical fitness, consistent with the findings of previous studies (61). The research findings further revealed that female students reared under an authoritative style of motherly parenting scored significantly higher in the 800-meter run and had a significantly better overall physical health score compared to those reared under an authoritarian style. This suggests that the authoritative style of parenting, which combines warmth and firmness, is also reasonable for promoting physical health development, aligning with the views of previous researchers (57, 58). However, it is crucial to note that previous studies have also found no significant differences between authoritative and authoritarian parenting styles in several physical health-related indicators. Therefore, it is imperative to refrain from transitioning from an authoritative parenting style to an authoritarian one, as the latter tends to be overly stringent and constraining, potentially resulting in undesirable consequences for a college student’s physical and mental well-being.

### 4.4 Association between parenting styles and males’ physical health

The results for male students indicated a positive correlation between the mother’s encouragement of autonomy and performance in the 1,000-meter run, pull-ups, and overall physical health levels. Conversely, the father’s encouragement factor exhibited a negatively correlated with pull-up performance. The mother’s encouragement of autonomy had a significant positive impact on the physical health of male students. When mothers encourage their children to think independently, make autonomous decisions, and provide appropriate support and guidance during their growth, it helps establish a positive attitude towards life and healthy habits. This encouraging approach may prompt male students to engage more actively in physical activities such as the 1,000-meter run and pull-ups, subsequently enhancing their physical health. However, in contrast to the mother’s influence, the father’s encouragement factor exhibited a negative correlation with the male students’ pull-up performance. This could be attributed to fathers potentially emphasizing outcomes over the process when encouraging their children, leading to a lack of patience and perseverance when facing challenges. Consequently, it may affect their performance in physically challenging activities like pull-ups. Furthermore, in numerous cultures, parents frequently harbor differing expectations for their sons and daughters, leading to distinct educational approaches tailored specifically for each gender (45). This gender-role difference may lead parents to adopt varying strategies and techniques when encouraging their children.

Further observations on the characteristics of physical health of college male students with different parenting styles. Specifically, those reared under a permissive motherly style scored the highest in the 50-meter run, significantly outperforming those under an authoritarian style. Additionally, students reared under a democratic motherly style demonstrated significantly better performance in standing long jump and sit-and-reach tests than those reared under the authoritarian style. Furthermore, the permissive style was associated with the best performance in the 1,000-meter run and pull-ups, with scores significantly higher than those of the authoritarian style. Overall, males reared under a permissive motherly style exhibited the highest total physical health score, significantly surpassing those under the authoritarian style. Turning to the influence of fatherly parenting styles, those reared under a democratic style demonstrated the best performance in the standing long jump, significantly outperforming both the authoritative and authoritarian styles. In addition, democratic parents had the highest total physical health scores, significantly higher than authoritarian parents. Taken together, democratic fathers and permissive mothers appear to be the ideal parenting model for promoting boys’ physical health. This finding contrasts with previous research, which has typically concluded that permissive and democratic parenting styles are most detrimental to adolescents’ physical and mental health (57, 58). This discrepancy could be attributed to differences in population characteristics and cultural environments. On the other hand, the authoritarian style emerged as the least favorable for physical health development, consistent with previous research (61).

### 4.5 Educational implications

The study discovered significant patterns regarding the connections between parenting styles and physical health of college students. Democratic parenting styles, both from mothers and fathers, are especially beneficial for females, boosting their physical health. In contrast, authoritarian and permissive styles indicate a negative effect. For males, democratic paternal parenting associated with a moderately permissive maternal approach emerge as the most effective strategies for nurturing healthy physical development. Conversely, authoritarian parenting stands out as a detrimental factor, adversely affecting males’ physical wellbeing. Existing research provided potential mechanisms for the important role of parental styles in physical health of the university students. Moè and Katz (62) raised awareness of the relationship between parents’ attitude and their children’s behaviors. Positive attitude of parents leads to positive emotions and high self-efficacy in tasks such as homework. This could be also applied to exercise-related behaviors of university students. Parents holding a positive attitude towards exercise encourage and facilitate children developing a physically active life style which tends to result in better physical health status over a long time period. In this logic, parental styles play the role as a moderator of physical health among the university students. This can be substantiated by evidence that each parental style is a moderator of adolescent socialization outcomes (63).

To shape more effective parenting, a series of strategies were proposed. For males: (1) Strengthening democratic father’s parenting: Fathers should be proactive in building a relationship with their sons based on mutual respect and understanding. By encouraging open communication and valuing their sons’ perspectives during the planning of sports activities or related endeavors, fathers can ignite a spark of interest in sports and foster an active engagement in physical exercise. Whenever males demonstrate progress or triumph over challenges in sports, fathers should promptly offer positive reinforcement, thereby bolstering their sense of accomplishment, self-esteem, and fostering a lifelong dedication to exercise, ultimately contributing to their physical well-being. (2) Moderate permissive mother’s parenting: A permissive parenting style by mothers, devoid of proper guidance, can potentially undermine males’ physical health by fostering a lack of self-discipline and responsibility, ultimately impeding the maintenance of healthy lifestyle habits. However, integrating reasonable guidance into this parenting approach, setting clear health goals and athletic expectations while simultaneously granting boys sufficient autonomy to achieve them, strikes a balance that is conducive. This equilibrium fosters self-motivated exercise routines in boys, reduces their reliance on external pressure, and thereby sustains their enthusiasm for sports over a longer period, ultimately promoting physical well-being.

For females: (1) Advocating dual democratic parenting by fathers and mothers: Encouraging both mothers and fathers to adopt a democratic parenting approach fosters a family atmosphere of mutual respect and understanding, granting females greater autonomy and choice in their lives. By supporting their engagement in sports based on personal interests, this parenting style is more likely to stimulate females’ intrinsic motivation, cultivate a positive attitude towards sports, and encourage them to actively choose and participate in physical activities, ultimately enhancing their overall physical health. (2) Strengthening authoritative mother’s parenting: Authoritative mothers, while providing love and support to their daughters, also establish clear rules and expectations. This parenting approach encourages self-discipline in sports, motivating girls to adhere to their exercise plans and effectively prevent health issues stemming from laziness or procrastination. Furthermore, authoritative mothers can set positive examples for their daughters through their own behaviors, such as engaging in regular physical activity and maintaining a healthy diet. In terms of common strategies, it is important to avoid an authoritarian approach to upbringing. This type of upbringing tends to restrict the freedom of choice and decision-making of university students, which can have a serious negative impact on their physical and mental health. Parents should abandon authoritarian parenting styles and refrain from excessive intervention in their children’s choice of sports. Instead, they should primarily guide and support their children, allowing them to discover suitable forms of exercise through exploration, thereby promoting both physical and mental health. At the same time, parents need to pay careful attention to their children’s emotional changes, reduce their psychological pressure through communication and understanding, and promote the formation of healthy exercise habits. In summary, through in-depth analysis of the effects of parenting styles on physical health, we reveal the complex mechanisms behind them and propose a series of practical strategies to optimize the family parenting environment in the context of gender differences. In the future, a comprehensive exploration of the mechanisms underlying how parenting styles impact physical health, mediated by physiological (heart rate, blood pressure, hormone levels) and psychological (stress levels, self-efficacy, exercise motivation) factors, is crucial. This endeavor will establish a firmer scientific foundation for refining family parenting strategies, ultimately aiding in fostering both physical and psychological wellbeing among children.

### 4.6 Limitations

Firstly, as a cross-sectional study, it is impossible to establish a causal relationship between physical health and parenting styles. Nevertheless, our research does reveal significant associations between parenting styles and physical well-being. Secondly, we relied on questionnaires to assess parenting styles. While the selected scales have gained widespread recognition among scholars, it’s crucial to note that the measured parenting styles do not equate to structured clinical diagnoses. Meanwhile, the parenting style evaluated through questionnaires is based on the subjective feelings and understanding of students, specifically, how they “perceive” the parenting style of their parents. This perception may deviate from the actual parenting style adopted by parents, which could potentially impact the accuracy and universality of the research findings. In the future, to enhance the rigor of the assessment, a more comprehensive approach that incorporates the joint participation of both children and parents could be adopted to evaluate parenting styles. Thirdly, the generalizability of our findings may be limited. Although the sample size was considerable, and participants came from various provinces, our data may not fully represent all Chinese college students or other populations. In addition, differences in culture, measurement tools, methods, and assessment criteria may also lead to differences in findings compared to other studies. It is noteworthy that our study provides valuable insights into the relationship between parenting styles and physical health among Chinese college students. However, future longitudinal studies are needed to further explore the causal mechanisms of these associations. Moreover, it would be beneficial to consider the influence of other potential factors, such as social support and environmental factors, to obtain a more comprehensive understanding of the factors that affect physical health. Despite the limitations, our study offers a foundation for future research in this area. It highlights the importance of considering parenting styles in promoting the physical well-being of college students.

## 5 Conclusion

This study provides evidence for the association between parental parenting behaviors and the physical health of college students from a non-Western perspective. Our findings reveal that parental warmth and encouragement of autonomy correlate positively with most physical health indicators, while excessive parental control is negatively associated with them. In other words, positive parenting styles contribute to improved physical health outcomes for children, while negative parenting styles may have adverse effects, particularly among female students. Furthermore, our results indicate that a democratic parenting style is most suitable for promoting the physical health of female students. For male students, however, combining a democratic fatherly style and a permissive motherly style appears to be more beneficial. This discovery underscores the importance of enhancing physical health through improved parenting practices and highlights the crucial role of adopting differentiated parenting approaches based on gender. Therefore, this study calls for increased attention to parenting styles in the field of public health and the implementation of relevant intervention programs aimed at further improving the physical health of the college population.

## Data Availability

The raw data supporting the conclusions of this article will be made available by the authors, without undue reservation.
