# Supplementary material for: Effects of different parenting styles on the physical health of Chinese university students
Source: Front Public Health. 2024 Aug 27;12:1433538. doi: 10.3389/fpubh.2024.1433538 (PMC11385859; doi:10.3389/fpubh.2024.1433538)
Supplement: Supplementary file 1 [file Table_1.DOCX]

Supplementary Material

# Supplementary Tables

# Appendix 1: Comparison of parenting style scores among different groups of college students (*N* = 3,151).

| Constituencies | **Gander** | |  | **Family members** | |  | **Hometown types** | |  | **Major types** | |
| --- | --- | --- | --- | --- | --- | --- | --- | --- | --- | --- | --- |
|  | Male | Female |  | One | More than one |  | City | Country |  | Social Sciences | Natural Sciences |
| *N* (%) | 1,365 (43.32) | 1,786 (56.68) |  | 1,785 (56.65) | 1,366 (43.35) |  | 1,723 (54.68) | 1,428 (45.32) |  | 1,469 (46.62) | 1,682 (53.38) |
| **PBI-Mother (Mean ± SD)** | | | | | | | | | | | |
| Care | 23.75 ± 4.60 | 23.85 ± 5.00 |  | 24.26 ± 4.73 | 23.21 ± 4.90 |  | 24.10 ± 4.84 | 23.45 ± 4.80 |  | 23.76 ± 4.97 | 23.85 ± 4.71 |
| t (*p*) –value | 0.59 (0.55) | |  | **6.07 (*p* < 0.01)** | |  | **-3.77 (*p* < 0.01)** | |  | -0.544 (0.59) | |
| Autonomy | 10.92 ± 3.61 | 11.38 ± 3.60 |  | 11.25 ± 3.62 | 11.10 ± 3.60 |  | 11.41 ± 3.69 | 10.91 ± 3.50 |  | 11.31 ± 3.63 | 11.07 ± 3.59 |
| t (*p*) –value | **3.55 (*p* < 0.01)** | |  | -1.21 (0.23) | |  | **-3.90 (*p* < 0.01)** | |  | 1.82 (0.07) | |
| Overprotection | 6.30 ± 2.73 | 6.14 ± 2.55 |  | 6.31 ± 2.71 | 6.07 ± 2.50 |  | 6.25 ± 2.71 | 6.15 ± 2.53 |  | 6.19 ± 2.62 | 6.23 ± 2.64 |
| t (*p*) –value | -1.69 (0.09) | |  | **-2.54 (*p* < 0.05)** | |  | -1.08 (0.28) | |  | -0.40 (0.69) | |
| **PBI-Father (Mean ± SD)** | | | | | | | | | | | |
| Care | 21.96 ± 5.22 | 22.59 ± 5.50 |  | 22.70 ± 5.37 | 21.82 ± 5.37 |  | 22.63 ± 5.41 | 21.94 ± 5.35 |  | 22.42 ± 5.47 | 22..23 ± 5.32 |
| t (*p*) -value | **3.27 (*p* < 0.01)** | |  | **-4.60 (*p* < 0.01)** | |  | **-3.59 (*p* < 0.01)** | |  | 1.00 (0.32） | |
| Autonomy | 11.92 ± 3.66 | 12.16 ± 3.70 |  | 12.29 ± 3.69 | 11.75 ± 3.65 |  | 12.37 ± 3.70 | 11.66 ± 3.62 |  | 12.18 ± 3.64 | 11.94 ± 3.72 |
| t (*p*) -value | 1.81 (0.07) | |  | **-4.07 (*p* < 0.01)** | |  | **-5.42 (*p* < 0.01)** | |  | 1.88 (0.06) | |
| Overprotection | 5.74 ± 2.59 | 5.79 ± 2.45 |  | 5.77 ± 2.55 | 5.77 ± 2.47 |  | 5.73 ± 2.54 | 5.82 ± 2.48 |  | 5.77 ± 2.46 | 5.77 ± 2.57 |
| t (*p*) -value | 0.52 (0.60) | |  | -0.09 (0.93) | |  | 0.94 (0.35) | |  | 0.04 (0.97) | |

**Appendix 2: Comparison of physical fitness scores among different groups of college students**.

| Constituencies | **Family members** | |  | **Hometown types** | |  | **Major types** | |
| --- | --- | --- | --- | --- | --- | --- | --- | --- |
|  | One | More than one |  | City | Country |  | Social Sciences | Natural Sciences |
| **Male's physical fitness (*N* = 1,365)** | | | | | | | | |
| BMI | 13.49 ± 1.99 | 13.79 ± 1.83 |  | 13.53 ± 1.99 | 13.68 ± 1.87 |  | 13.55 ± 1.85 | 13.62 ± 1.96 |
| t (*p*) -value | **-2.88 (*p* < 0.01)** | |  | -1.40 (0.16) | |  | -0.58 (0.56) | |
| Lung capacity | 11.67 ± 2.64 | 11.06 ± 2.90 |  | 11.67 ± 2.79 | 11.19 ± 2.71 |  | 10.99 ± 3.05 | 11.56 ± 2.66 |
| t (*p*) -value | **3.97 (*p* < 0.001)** | |  | **3.22 (*p* < 0.01)** | |  | **-2.89 (*p* < 0.01)** | |
| 50-m sprint | 14.96 ± 1.97 | 14.91 ± 2.02 |  | 15.00 ± 2.01 | 14.88 ± 1.97 |  | 14.79 ± 2.01 | 14.98 ± 1.98 |
| t (*p*) -value | 0.48 (0.63) | |  | 1.13 (0.26) | |  | -1.46 (0.14) | |
| Standing long jump | 6.10 ± 1.92 | 6.41 ± 1.56 |  | 6.15 ± 1.88 | 6.29 ± 1.70 |  | 6.15 ± 1.75 | 6.24 ± 1.81 |
| t (*p*) -value | **-3.23 (*p* < 0.01)** | |  | -1.41 (0.16) | |  | -0.71 (0.47) | |
| 1000-m run | 11.19 ± 3.08 | 11.83 ± 2.75 |  | 11.37 ± 3.06 | 11.50 ± 2.88 |  | 11.24 ± 3.01 | 11.49 ± 2.96 |
| t (*p*) -value | **-4.01 (*p* < 0.001)** | |  | -0.79 (0.43) | |  | -1.24 (0.21) | |
| Seated forward bend | 6.40 ± 2.15 | 6.75 ± 2.03 |  | 6.39 ± 2.12 | 6.69 ± 2.09 |  | 6.47 ± 2.00 | 6.55 ± 2.14 |
| t (*p*) -value | **-2.97 (*p* < 0.01)** | |  | **-2.60 (*p* < 0.01)** | |  | -0.58 (0.56) | |
| Pull ups | 0.37 ± 1.17 | 0.69 ± 1.64 |  | 0.37 ± 1.15 | 0.62 ± 1.58 |  | 0.37 ± 1.06 | 0.53 ± 1.45 |
| t *(p*) -value | **-3.85 (*p* < 0.001)** | |  | **-3.39 (*p* < 0.01)** | |  | **-2.04 (*p* < 0.05)** | |
| Physical fitness score | 64.19 ± 7.41 | 65.43 ± 7.22 |  | 64.49 ± 7.55 | 64.85 ± 7.16 |  | 63.57 ± 7.62 | 64.96 ± 7.27 |
| t (*p*) -value | **-3.06 (*p* < 0.01)** | |  | -0.90 (0.37) | |  | **-2.89 (*p* < 0.01)** | |
| **Female's physical fitness (*N* = 1,786)** | | | | | | | | |
| BMI | 14.34 ± 1.42 | 14.42 ± 1.33 |  | 14.35 ± 1.39 | 14.41 ± 1.37 |  | 14.39 ± 1.37 | 14.35 ± 1.41 |
| t (*p*) -value | -1.14 (0.25) | |  | -0.88 (0.38) | |  | 0.68 (0.50) | |
| Lung capacity | 11.97 ± 1.88 | 11.52 ± 1.75 |  | 11.94 ± 1.84 | 11.53 ± 1.80 |  | 11.74 ± 1.82 | 11.80 ± 1.87 |
| t (*p*) -value | **5.24 (*p* < 0.001)** | |  | **4.71 (*p* < 0.001)** | |  | -0.58 (0.56) | |
| 50-m run | 13.25 ± 2.48 | 12.87 ± 2.95 |  | 13.25 ± 2.59 | 12.82 ± 2.85 |  | 12.94 ± 2.80 | 13.33 ± 2.52 |
| t (*p*) -value | **2.87 (*p* < 0.01)** | |  | **3.28 (*p* < 0.01)** | |  | **-3.01 (*p* < 0.01)** | |
| Standing-long-jump | 6.62 ± 1.11 | 6.87 ± 1.10 |  | 6.70 ± 1.13 | 6.78 ± 1.09 |  | 6.68 ± 1.09 | 6.84 ± 1.16 |
| t (*p*) -value | **-4.83 (*p* < 0.001)** | |  | -1.58 (0.11) | |  | **-2.83 (*p* < 0.01)** | |
| 800-m run | 12.18 ± 3.13 | 12.63 ± 2.84 |  | 12.37 ± 3.12 | 12.41 ± 2.84 |  | 12.27 ± 2.99 | 12.61 ± 3.02 |
| t (*p*) -value | **-3.19 (*p* < 0.01)** | |  | 0.29 (0.77) | |  | **-2.26 (*p* < 0.05)** | |
| Seated forward bend | 6.92 ± 1.85 | 7.18 ± 1.76 |  | 6.93 ± 1.97 | 7.21 ± 1.70 |  | 7.07 ± 1.86 | 6.99 ± 1.87 |
| t (*p*) -value | **-2.97 (*p* < 0.01)** | |  | **-3.15 (*p* < 0.01)** | |  | 0.82 (0.41) | |
| Bent-leg-sit-ups | 6.38 ± 1.26 | 5.98 ± 1.23 |  | 6.38 ± 1.23 | 5.92 ± 1.25 |  | 6.13 ± 1.23 | 6.30 ± 1.32 |
| t (*p*) -value | **6.77 (*p* < 0.001)** | |  | **7.72 (*p* < 0.001)** | |  | **-2.60 (*p* < 0.01)** | |
| Physical fitness score | 71.64 ± 6.27 | 71.47 ± 5.97 |  | 71.91 ± 6.25 | 71.08 ± 5.93 |  | 71.22 ± 6.17 | 72.21 ± 6.00 |
| t (*p*) -value | 0.61 (0.54) | |  | **2.85 (*p* < 0.01)** | |  | **-3.25 (*p* < 0.01)** | |

**Appendix 3：Correlation between parenting styles and physical health among college students.**

| Pearson | Male (*N* = 1,365) | | | | | |  | Female (*N* = 1,786) | | | | | |
| --- | --- | --- | --- | --- | --- | --- | --- | --- | --- | --- | --- | --- | --- |
|  | M-C | M-A | M-O | F-C | F-A | F-O |  | M-C | M-A | M-O | F-C | F-A | F-O |
| BMI | **0.057*** | **0.087**** | -0.052 | 0.041 | **0.076**** | -0.014 |  | **0.053*** | 0.039 | **-0.057*** | **0.081**** | 0.032 | -0.035 |
| Lung capacity | 0.019 | **0.063*** | -0.005 | 0.029 | **0.069*** | -0.006 |  | **0.118**** | **0.063**** | **-0.064**** | **0.074**** | **0.112**** | **-0.052*** |
| 50-m sprint | **0.059*** | **0.109**** | **-0.097**** | 0.043 | **0.093**** | **-0.085**** |  | **0.154**** | **0.153**** | **-0.059*** | **0.075**** | **0.093**** | **-0.058*** |
| Standing long jump | **0.070**** | **0.116**** | **-0.111**** | **0.080**** | **0.100**** | **-0.082**** |  | **0.062**** | **0.109**** | **-0.073**** | 0.017 | **0.071**** | **-0.072**** |
| Seated forward bend | **0.071**** | **0.074**** | **-0.059*** | 0.052 | **0.065*** | -0.043 |  | 0.021 | **0.079**** | -0.040 | 0.020 | **0.075**** | -0.037 |
| 1000-m run | **0.072**** | **0.164**** | **-0.054*** | **0.066*** | **0.080**** | -0.042 |  | **0.122**** | **0.136**** | **-0.140**** | **0.098**** | **0.094**** | **-0.095**** |
| Pull ups / Bent-leg-sit-ups | **0.057*** | **0.100**** | **-0.068*** | 0.047 | 0.023 | -0.050 |  | **0.123**** | **0.078**** | **-0.049*** | **0.056*** | **0.049*** | **-0.055*** |
| Physical fitness score | **0.115**** | **0.210**** | **-0.120**** | **0.103**** | **0.151**** | **-0.088**** |  | **0.219**** | **0.222**** | **-0.162**** | **0.143**** | **0.174**** | **-0.132**** |

**Note:** “M-C = Mother Care”; “M-A = Mother Autonomy”; “M-O = Mother Overprotection”; “F-C = Father Care”; “F-A = Father Autonomy”; “F-O = Father Overprotection”; ** Significant correlation at 0.01 level (two-tailed); * Significant correlation at 0.05 level (two-tailed).

**Appendix 4: Comparison of scores related to females' physical fitness across different types of mother's parenting styles (Mean ± SD).**

| Constituencies | **Au-T (n = 304)** |  | **A-T (n = 451)** |  | **D-T (n = 744)** |  | **L-T (n = 287)** |  | ***F*** | ***p*** |
| --- | --- | --- | --- | --- | --- | --- | --- | --- | --- | --- |
| **BMI** | 14.41 ± 1.34 |  | 14.16 ± 1.61 |  | 14.46 ± 1.27 |  | 14.48 ± 1.27 |  | **5.366** | **0.001**** |
| **Lung capacity** | 11.78 ± 1.84 |  | 11.54 ± 1.73 |  | 11.98 ± 1.91 |  | 11.54 ± 1.72 |  | **7.031** | **0.000**** |
| **50-m run** | 13.30 ± 2.11 |  | 12.76 ± 3.12 |  | 13.31 ± 2.39 |  | 12.70 ± 3.25 |  | **6.487** | **0.000**** |
| **Standing-long-jump** | 6.63 ± 1.10 |  | 6.66 ± 1.09 |  | 6.83 ± 1.10 |  | 6.71 ± 1.20 |  | **3.55** | **0.014*** |
| Seated forward bend | 7.01 ± 1.79 |  | 6.94 ± 1.94 |  | 7.14 ± 1.81 |  | 6.99 ± 1.98 |  | 1.287 | 0.277 |
| **800-m run** | 12.39 ± 2.74 |  | 11.71 ± 3.43 |  | 12.81 ± 2.71 |  | 12.34 ± 3.12 |  | **12.748** | **0.000**** |
| **Bent-leg-sit-ups** | 6.25 ± 1.10 |  | 6.05 ± 1.25 |  | 6.31 ± 1.27 |  | 6.02 ± 1.37 |  | **6.143** | **0.000**** |
| **Physical fitness score** | 71.76 ± 5.17 |  | 69.81 ± 6.44 |  | 72.84 ± 5.84 |  | 70.78 ± 6.53 |  | **25.842** | **0.000**** |

**Note:** "Au-t = Authoritative type"; "A-T = Autocratic type"; "D-T = Democratic type"; "L-T = Laissez-faire type"; * *p* < 0.05; ** *p* < 0.01.

**Appendix 5: Comparison of scores related to females' physical fitness across different types of father's parenting styles (Mean ± SD).**

| Constituencies | **Au-T (n = 361)** |  | **A-T (n = 554)** |  | **D-T (n = 609)** |  | **L-T (n = 262)** |  | ***F*** | ***p*** |
| --- | --- | --- | --- | --- | --- | --- | --- | --- | --- | --- |
| BMI | 14.42 ± 1.31 |  | 14.30 ± 1.47 |  | 14.47 ± 1.27 |  | 14.27 ± 1.51 |  | 2.281 | 0.078 |
| **Lung capacity** | 11.80 ± 1.85 |  | 11.62 ± 1.77 |  | 11.93 ± 1.87 |  | 11.60 ± 1.82 |  | **3.568** | **0.014*** |
| **50-m run** | 13.10 ± 2.39 |  | 12.77 ± 3.14 |  | 13.26 ± 2.45 |  | 13.22 ± 2.69 |  | **3.543** | **0.014*** |
| Standing-long-jump | 6.69 ± 1.07 |  | 6.65 ± 1.13 |  | 6.80 ± 1.10 |  | 6.81 ± 1.16 |  | 2.405 | 0.066 |
| Seated forward bend | 6.92 ± 1.86 |  | 6.96 ± 1.97 |  | 7.17 ± 1.78 |  | 7.10 ± 1.86 |  | 1.85 | 0.136 |
| **800-m run** | 12.48 ± 2.75 |  | 11.92 ± 3.29 |  | 12.79 ± 2.75 |  | 12.32 ± 3.16 |  | **8.282** | **0.000**** |
| Bent-leg-sit-ups | 6.19 ± 1.20 |  | 6.09 ± 1.25 |  | 6.26 ± 1.34 |  | 6.24 ± 1.16 |  | 2.045 | 0.106 |
| **Physical fitness score** | 71.60 ± 5.21 |  | 70.30 ± 6.55 |  | 72.69 ± 6.00 |  | 71.55 ± 6.19 |  | **15.04** | **0.000**** |

**Note:** "Au-t = Authoritative type"; "A-T = Autocratic type"; "D-T = Democratic type"; "L-T = Laissez-faire type"; * *p* < 0.05; ** *p* < 0.01.

**Appendix 6: Comparison of scores related to males' physical fitness across different types of mother's parenting styles (Mean ± SD).**

| Constituencies | **Au-T (n=218)** |  | **A-T (n=409)** |  | **D-T (n=547)** |  | **L-T (n=191)** |  | ***F*** | ***p*** |
| --- | --- | --- | --- | --- | --- | --- | --- | --- | --- | --- |
| BMI | 13.53 ± 1.96 |  | 13.45 ± 2.02 |  | 13.67 ± 1.91 |  | 13.84 ± 1.79 |  | 2.079 | 0.101 |
| Lung capacity | 11.48 ± 2.95 |  | 11.40 ± 2.83 |  | 11.50 ± 2.62 |  | 11.26 ± 2.78 |  | 0.386 | 0.763 |
| **50-m run** | 14.94 ± 1.51 |  | 14.68 ± 1.97 |  | 15.05 ± 2.23 |  | 15.22 ± 1.69 |  | **4.127** | **0.006**** |
| **Standing-long-jump** | 6.12 ± 1.93 |  | 5.93 ± 1.84 |  | 6.41 ± 1.72 |  | 6.40 ± 1.66 |  | **6.587** | **0.000**** |
| Seated forward bend | 6.53 ± 1.92 |  | 6.33 ± 2.19 |  | 6.69 ± 2.11 |  | 6.55 ± 2.13 |  | 2.343 | 0.072 |
| **1000-m run** | 11.42 ± 3.02 |  | 11.07 ± 2.86 |  | 11.61 ± 3.00 |  | 11.72 ± 3.02 |  | **3.304** | **0.020*** |
| **Pull-ups** | 0.52 ± 1.32 |  | 0.35 ± 1.16 |  | 0.51 ± 1.43 |  | 0.74 ± 1.69 |  | **3.593** | **0.013*** |
| **Physical fitness score** | 64.54 ± 7.44 |  | 63.21 ± 6.72 |  | 65.43 ± 7.63 |  | 65.72 ± 7.35 |  | **8.826** | **0.000**** |

**Note:** "Au-t = Authoritative type"; "A-T = Autocratic type"; "D-T = Democratic type"; "L-T = Laissez-faire type"; * *p* < 0.05; ** *p* < 0.01.

**Appendix 7: Comparison of scores related to males' physical fitness across different types of mother's parenting styles (Mean ± SD).**

| Constituencies | **Au-t (n = 215)** |  | **A-t (n = 466)** |  | **D-T (n = 510)** |  | **L-T (n = 174)** |  | ***F*** | ***p*** |
| --- | --- | --- | --- | --- | --- | --- | --- | --- | --- | --- |
| BMI | 13.63 ± 1.98 |  | 13.51 ± 1.99 |  | 13.70 ± 1.85 |  | 13.55 ± 1.98 |  | 0.871 | 0.456 |
| Lung capacity | 11.36 ± 3.21 |  | 11.45 ± 2.71 |  | 11.49 ± 2.59 |  | 11.35 ± 2.79 |  | 0.180 | 0.910 |
| 50-m run | 14.81 ± 2.37 |  | 14.80 ± 1.78 |  | 15.12 ± 2.00 |  | 14.96 ± 1.95 |  | 2.441 | 0.063 |
| **Standing-long-jump** | **6.05 ± 2.03** |  | **6.09 ± 1.80** |  | **6.45 ± 1.61** |  | **6.09 ± 1.95** |  | **4.616** | **0.003**** |
| Seated forward bend | 6.54 ± 2.10 |  | 6.40 ± 2.14 |  | 6.67 ± 2.09 |  | 6.49 ± 2.09 |  | 1.415 | 0.237 |
| 1000-m run | 11.40 ± 3.21 |  | 11.20 ± 2.79 |  | 11.63 ± 3.08 |  | 11.55 ± 2.77 |  | 1.808 | 0.144 |
| Pull-ups | 0.59 ± 1.47 |  | 0.37 ± 1.15 |  | 0.58 ± 1.52 |  | 0.49 ± 1.37 |  | 2.242 | 0.082 |
| **Physical fitness score** | **64.37 ± 8.41** |  | **63.81 ± 6.54** |  | **65.63 ± 7.45** |  | **64.48 ± 7.52** |  | **5.237** | **0.001**** |

**Note:** "Au-t = Authoritative type"; "A-T = Autocratic type"; "D-T = Democratic type"; "L-T = Laissez-faire type"; * *p* < 0.05; ** *p* < 0.01.
